# Prevalence of cardiovascular drug-related adverse drug reactions consultations in UK primary care: A cross-sectional study

**DOI:** 10.1371/journal.pone.0307237

**Published:** 2024-07-24

**Authors:** Widya N. Insani, Cate Whittlesea, Li Wei

**Affiliations:** 1 Department of Pharmacology and Clinical Pharmacy, Padjadjaran University, Bandung, Indonesia; 2 Research Department of Practice and Policy, School of Pharmacy, University College London, London, United Kingdom; 3 Centre of Excellence for Pharmaceutical Care Innovation, Padjadjaran University, Bandung, Indonesia; 4 Centre for Medicines Optimisation Research and Education, University College London Hospitals NHS Foundation Trust, London, United Kingdom; HT Ong Heart Clinic, MALAYSIA

## Abstract

**Background:**

Adverse drug reactions (ADRs) represent a significant barrier to achieve optimal treatment outcomes. Cardiovascular drugs, including antihypertensive drugs, lipid-lowering drugs, and antithrombotic drugs, are among the most prescribed medications in the primary care setting.

**Objectives:**

To estimate the prevalence of cardiovascular drug-related ADRs consultations in United Kingdom (UK) primary care and identify risk factors of these ADRs.

**Methods:**

This was a cross-sectional study of cardiovascular drug users between 2000–2019 using UK IQVIA Medical Research Data. ADRs consultations were identified using database screening method employing standardised designated codes. The overall and annual age-standardised prevalence was estimated using direct standardisation method using 2019 mid-year UK population. Risk factors of ADRs consultations were estimated using logistic regression model stratified by therapeutic areas.

**Results:**

The standardised prevalence of consultations related to cardiovascular drugs ADRs was 10.60 (95% CI. 10.46, 10.75) per 1000 patients. Patients aged 70–79 years had the highest occurrence of ADRs consultations. The most frequently drug classes implicated in the ADRs consultations were statins (n = 9,993 events, 27.09%), beta-blockers (n = 8,538 events, 23.15%), ACEIs/ARBs (n = 8,345 events, 22.62%), and aspirin (n = 6,482 events, 17.57%). Risk factors of ADRs consultations were previous history of cardiovascular diseases, e.g., myocardial infarction and stroke; advanced age, comorbidities; diabetes and dyslipidaemia; and polypharmacy.

**Conclusions:**

The burden of cardiovascular drug-related ADRs consultations in primary care was considerable. Statins, beta-blockers, ACEIs/ARBs, and aspirin were the most frequently implicated drug classes. Closer clinical monitoring should be performed for patients affected by the ADRs to mitigate the risk of suboptimal treatment outcomes.

## Introduction

Cardiovascular diseases (CVD) are the leading cause of mortality and a major contributor to disability worldwide [1]. Cardiovascular drugs, including antihypertensive drugs, lipid-lowering drugs, and antithrombotic drugs are the cornerstone for the prevention and treatment of CVD with an established effectiveness in reducing the risk of CVD events and mortality [2–4]. Recent National Prescription Analysis (NPA) in England showed that cardiovascular drugs were the most frequently dispensed medication class, accounting for 29% of all items dispensed in 2022–2023 [5].

Adverse drug reactions (ADRs) represent a significant barrier in cardiovascular treatment, as they can lead to treatment discontinuation, non-adherence, or the need for additional medications to manage the symptoms [6–9]. Patients on cardiovascular drugs are particularly vulnerable to ADRs as these patients may have an altered metabolism, advanced age, comorbidities, and concomitant medications [10]. Cardiovascular drugs were among the most implicated drugs causing ADRs-related hospital admissions, e.g., major bleeding related with antithrombotic drugs and electrolyte imbalance due to antihypertensive drugs [11–13]. ADRs-related to cardiovascular drugs constituted 18.1%-42.9% of all ADRs reported among hospital inpatients [14–16].

In contrast, there is less information on the burden of cardiovascular drug-related ADRs in primary care. Primary care providers act as a gatekeeper and plays a crucial role in early recognition of ADRs to prevent further iatrogenic complications [17]. A previous study by Tsang et al showed that ADRs consultations occurred in 1.26 per 1000 general practice (GP) consultations in the UK, with cardiovascular drugs were the most drug classes implicated in the ADRs [18]. However, this study only captured one year data in 2007. A single practice-based study in Scotland showed that ADRs consultations constituted 1.7% of total GP consultations, with antihypertensive drugs were among the most frequently implicated medication [19]. There is a need to improve understanding on the burden of cardiovascular drugs-related ADRs consultations in the primary care setting as routine patient monitoring for CVD-related condition, such as hypertension and dyslipidaemia are largely conducted in this setting [20]. This understanding would help in prioritising therapeutic areas requiring intervention and may provide the basis for further investigation on the burden of ADRs for patients and the health system.

## Methods

### Data source

This study was conducted using IQVIA Medical Research Data UK that incorporates data from The Health Improvement Network (THIN), a Cegedim database [21]. The data contains de-identified information provided by patients as part of their routine primary care. Specific approval for the use of this data in the current submission was obtained from IMRD Scientific Review Committee with the project entitled: “*Impact of Adverse Drug Reactions of Cardiovascular Drugs on Patients Clinical Outcomes in the Primary Care Setting”* (reference number:21SRC008). The data were accessed for research purposes on April 2023.

### Study design

This was a cross-sectional study investigating the prevalence of consultations related to cardiovascular drugs ADRs in UK primary care setting. Patients who were newly prescribed cardiovascular drugs during 2000–2019 were included. Patients with invalid medical records (missing date of birth, sex, invalid registration date), aged <18 years at the first of any cardiovascular drug prescriptions and registered less than 6 months from the index date were excluded. Cardiovascular drugs included were i) angiotensin- converting enzyme inhibitors (ACEIs)/angiotensin receptor blocker (ARBs), ii) calcium channel blockers (CCBs), iii) diuretics, iv) lipid regulating drugs, v) antiplatelets and anticoagulants vi) beta blockers, and vii) nitrates [22]. ADRs consultations related with cardiovascular drugs were identified using standardised designated codes in primary care records, which has been previously validated [7, 8, 18, 23].

### Study variables

Several demographic and clinical characteristics were extracted for each patient. The variables included were sex, age, comorbidities (recorded at any time before or at the index date), i.e., history of established CVD defined as previous diagnosis of coronary heart disease (myocardial infarction (MI), angina), cerebrovascular disease (stroke and transient ischaemic attack (TIA)), peripheral vascular disease (PVD), and heart failure (HF) [24]; dyslipidaemia, hypertension, type 1 and type 2 diabetes mellitus, chronic kidney disease (CKD), liver disease, chronic obstructive pulmonary disease (COPD), rheumatic disease; and concomitant medications (recorded ≤ 180 days), including ACEIs/ARBs, CCBs, diuretics, beta‐blockers, antiplatelets and anticoagulants, antidiabetics, NSAIDs, and the presence of polypharmacy, defined as the use ≥ 5 medications [25].

### Data analysis

Patient characteristics were presented as numbers (percentage) for categorical variables and as means (±SD) for continuous variables. Overall crude prevalence was calculated by dividing the number of individuals with a cardiovascular drug-related ADR consultation by the number of patients with a prescription of the cardiovascular drug. Overall and annual age-standardized prevalence was calculated using direct standardisation method using mid-year UK population estimate for 2019 [26, 27]. For annual prevalence, the number of patients with a prescription of cardiovascular drug until 1 July each year was used as a denominator. The prevalence was expressed per 1000 patients. Risk factors of ADRs-related consultations were estimated using logistic regression model, stratified by therapeutic areas, i.e., antihypertensive, lipid-lowering, and antithrombotic drugs. Odds ratio (OR) and 95% confidence interval were presented. P-value less than 0.05 was considered statistically significant. Analyses were performed using SAS 9.4.

## Results

During the study period, 2,741,114 patients were prescribed at least one cardiovascular drug. After excluding patients who had invalid medical records (n = 116,732), younger than 18 years (n = 44,504), and registered in the general practices for less than 6 months (n = 843,927), the eligible patients in our analysis were 1,735,951 patients. About 10.49% (n = 182,058) of the patients had an established CVD (angina, MI, stroke/TIA, PVD, HF) before or at the first prescription any cardiovascular drug. The mean age of patients during the first prescription of any cardiovascular drugs was 53.33 (±16.49) in the CVD primary prevention (i.e., had no CVD event before the index date) cohort and 64.00 (±13.56) in the CVD secondary prevention cohort (had CVD before the index date) cohort.

### Prevalence of consultations related to cardiovascular drugs ADRs

**Fig 1:**
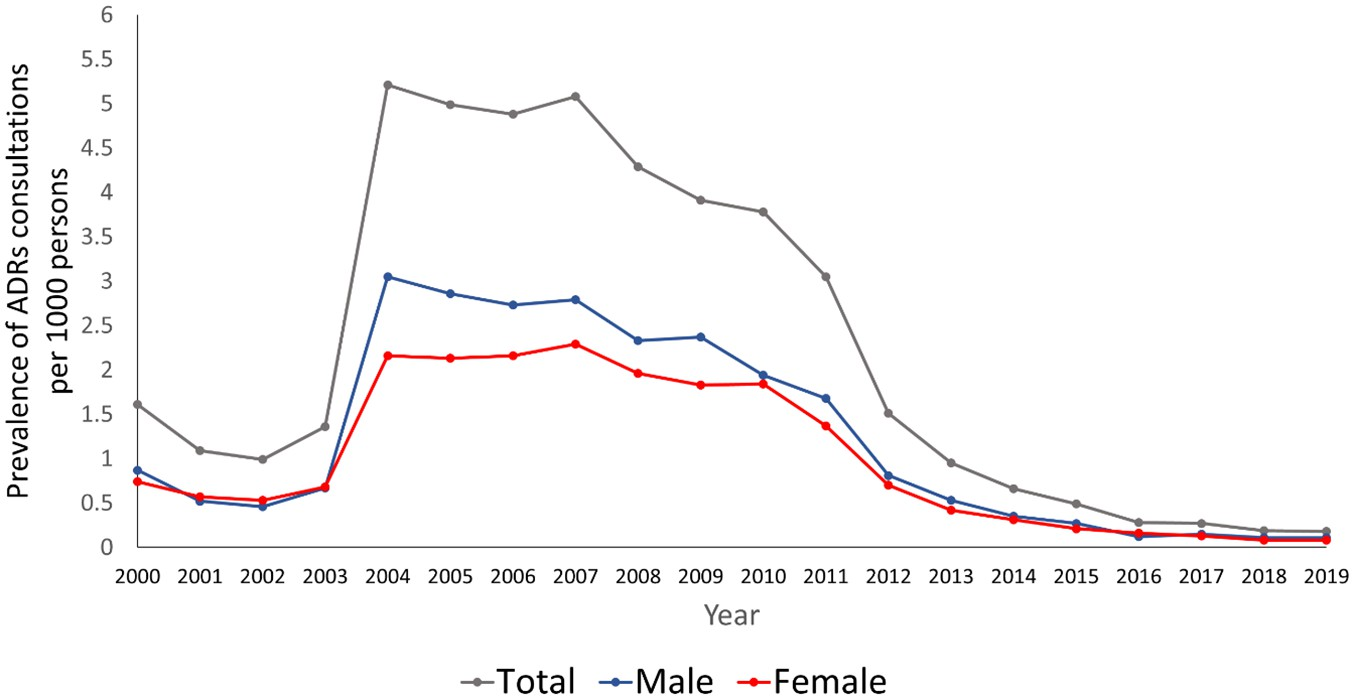
Temporal trend of consultations related to cardiovascular drugs ADRs during the study period.

The overall crude prevalence of consultations related to cardiovascular drugs ADRs was 13.50 (95 CI. 13.30, 13.70) per 1000 patients. The overall age-standardised prevalence was 10.60 (95% CI. 10.46, 10.75) per 1000 patients. The prevalence of ADRs consultations among male was 5.45 (95% CI. 5.35, 5.55), while that of female was 4.62 (95% CI. 4.53, 4.72) per 1000 patients. The standardised prevalence of ADRs consultations over time during the study period is presented in Fig 1.

**Fig 2:**
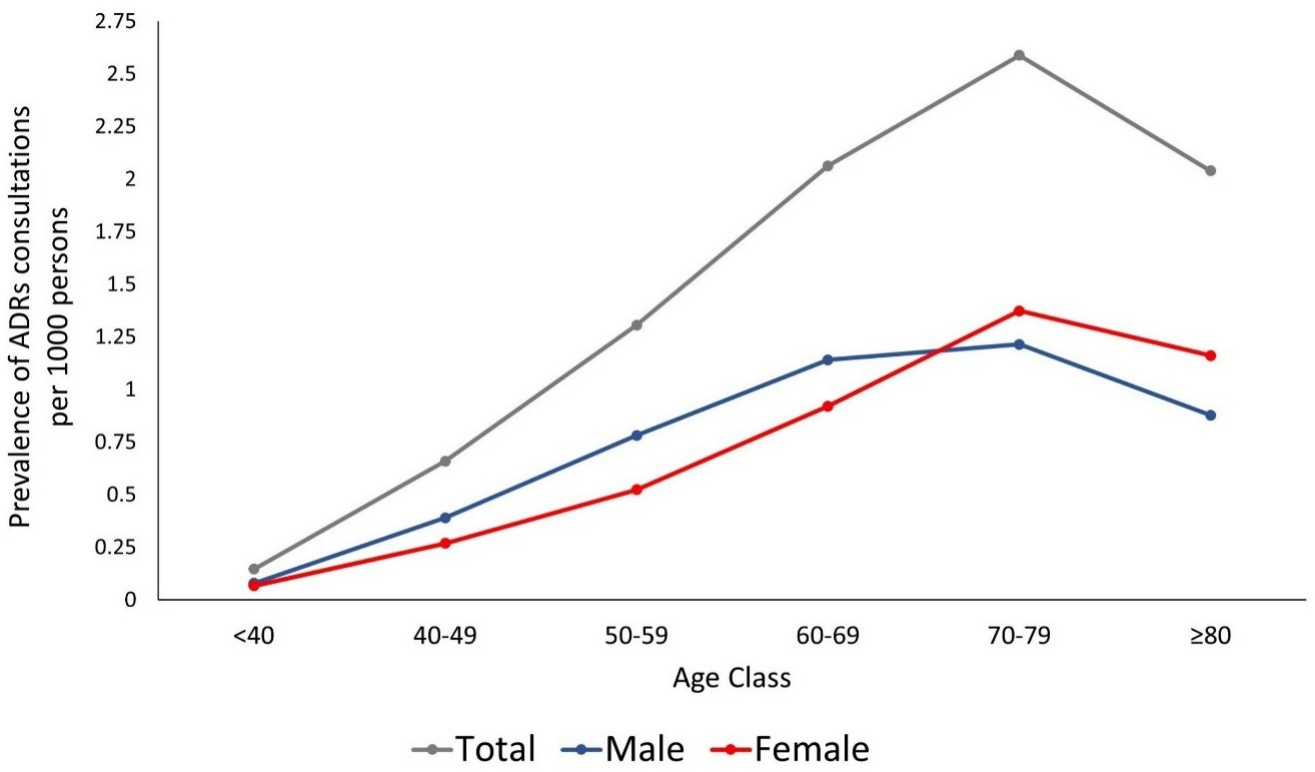
The burden of consultations related to cardiovascular drugs ADRs per age group.

Patients aged 70–79 years had the highest occurrence of cardiovascular drugs-related ADRs consultation, with the prevalence of 2.59 (95% CI. 2.52, 2.65) per 1000 patients. The lowest prevalence was observed among patients younger than 40. There was a decrease in ADRs occurrence among patients ≥ 80 years old (Fig 2). The most frequently drug classes implicated in the ADRs consultations were statins (n = 9,993 events, 27.09%), beta-blockers (n = 8,538 events, 23.15%), ACEIs/ARBs (n = 8,345 events, 22.62%), and aspirin (n = 6,482 events, 17.57%). The remaining proportion of 9.57% events were from other CVD drugs.

### Risk factors of ADRs consultations

Risk factors of ADRs consultations related to antihypertensive drugs, lipid lowering drugs, and antithrombotic drugs are presented in Tables 1–3, respectively. The strongest predictors of antihypertensive related ADR consultations included the advanced age, an established CVD (adjusted OR 3.49, 95% CI, 3.33, 3.65), the concomitant use of antithrombotic drugs (adjusted OR 1.65, 95% CI, 1.57, 1.74), and diabetes (adjusted OR 1.61, 95% CI, 1.52, 1.71). For ADRs related to lipid lowering drugs, the most significant risk factors included advanced age, diabetes (adjusted OR 2.06, 95% CI, 1.96, 2.18), an established CVD (adjusted OR. 1.46, 95% CI 1.38, 1.54), being female (adjusted OR 0.68, 95% CI, 0.65, 0.72), and rheumatic disease (adjusted OR 1.20, 95% CI, 1.11, 1.31). The risk factors of antithrombotic-related ADRs included advanced age, an established CVD (2.93, 95% CI. 2.74, 3.13), diabetes (1.30, 95% CI 1.19, 1.43), dyslipidaemia (1.25, 95% CI, 1.13, 1.38), and polypharmacy (1.16, 95% CI 1.08, 1.25).

**Table 1:** Risk factors of antihypertensive drugs-related ADRs consultations.

Variables	Patient with	Patients without	Crude Odds Ratio (95% CI)	P-value	Adjusted Odds Ratio (95% CI)	P-value
	ADR consultation	ADR consultation				
	n = 10,964 (100%)	n = 1,406,929 (100%)				
Sex (Male)	6,327 (57.71)	645, 799 (45.90)	1.61 (1.55, 1.67)	0.000	1.12 (1.08, 1.17)	0.000
Age (Mean ± SD)	64.68 (12.13)	54.50 (17.02)				
< 40 years	222 (2.02)	280,727 (19.95)	Reference		Reference	
40 ≤ age ≤ 49	987 (9.00)	244,350 (17.37)	5.11 (4.42, 5.91)	0.000	4.08 (3.53, 4.72)	0.000
50 ≤ age ≤ 59	2,273 (20.73)	302,420 (21.50)	9.50 (8.28, 10.91)	0.000	6.30 (5.48, 7.24)	0.000
60 ≤ age ≤ 69	3,276 (29.88)	276,141 (19.63)	15.00 (13.09, 17.19)	0.000	8.43 (7.34, 9.67)	0.000
70 ≤ age ≤ 79	2,888 (26.34)	196,393 (13.96)	18.60 (16.22, 21.32)	0.000	9.56 (8.32, 10.99)	0.000
≥ 80 years	1,318 (12.02)	106,898 (98.78)	15.59 (13.52, 17.98)	0.000	7.85 (6.79, 9.07)	0.000
Comorbidities						
CVD	5,066 (46.21)	171,171 (12.17)	6.20 (5.97, 6.44)	0.000	3.49 (3.33, 3.65)	0.000
Dyslipidaemia	997 (9.09)	71,997 (5.12)	1.85 (1.74, 1.98)	0.000	1.10 (1.03, 1.18)	0.000
Diabetes	1,490 (13.59)	98,728 (7.02)	2.08 (1.97, 2.20)	0.000	1.61 (1.52, 1.71)	0.000
CKD	372 (3.39)	32,035 (2.28)	1.51 (1.36, 1.67)	0.000	0.99 (0.89, 1.12)	0.922
Liver disease	126 (1.15)	22,296 (1.58)	0.72 (0.61, 0.86)	0.000	0.57 (0.44, 0.74)	0.000
COPD	1,083 (9.88)	79,699 (5.66)	1.83 (1.71, 1.94)	0.010	1.19 (1.12, 1.27)	0.000
Rheumatic disease	701 (6.39)	65,600 (4.66)	1.40 (1.29, 1.51)	0.000	0.95 (0.88, 1.03)	0.209
Concomitant Medications						
Lipid lowering drugs	3,994 (36.43)	211,942 (15.06)	3.23 (3.11, 3.36)	0.000	1.02 (0.97, 1.08)	0.343
Antiplatelets/ anticoagulants	4,363 (39.79)	183,475 (13.04)	4.41 (4.24, 4.58)	0.000	1.65 (1.57, 1.74)	0.000
NSAIDs	1,953 (17.81)	227,467 (16.17)	1.12 (1.07, 1.18)	0.017	1.13 (1.07, 1.19)	0.000
Polypharmacy (≥ 5 medications)	5,631 (51.36)	468,507 (33.30)	2.11 (2.04, 2.20)	0.000	1.03 (0.00, 1.08)	0.153

ADR: Adverse drug reaction. CVD: Cardiovascular Disease. CKD: Chronic kidney disease. COPD: Chronic obstructive pulmonary disease. NSAID: Non-steroidal anti-inflammatory drug.

**Table 2:** Risk factor of lipid lowering drugs-related ADRs consultations.

Variables	Patient with	Patients without ADR consultation	Crude Odds Ratio (95% CI)	P-value	Adjusted Odds Ratio (95% CI)	P-value
	ADR consultation					
	n = 7,712 (100%)	N = 748,461 (100%)				
Sex (Male)	3,731 (48.38)	432,780 (57.82)	0.68 (0.65, 0.72)	0.000	0.69 (0.66, 0.72)	0.000
Age (Mean ± SD)	64.16 (10.79)	60.98 (12.14)				
< 40 years	108 (1.40)	28,602 (3.82)	Reference		Reference	
40 ≤ age ≤ 49	582 (7.55)	95,003 (12.69)	1.62 (1.32, 1.99)	0.000	1.64 (1.33, 2.01)	0.000
50 ≤ age ≤ 59	1,655 (21.46)	194,856 (26.03)	2.25 (1.85, 2.73)	0.000	2.31 (1.90, 2.81)	0.000
60 ≤ age ≤ 69	2,751 (35.67)	234,401 (31.32)	3.11 (2.56, 3.77)	0.000	3.18 (2.62, 3.87)	0.000
70 ≤ age ≤ 79	1,997 (25.89)	146,147 (19.53)	3.62 (2.98, 4.39)	0.000	3.45 (2.83, 4.19)	0.000
≥ 80 years	619 (8.03)	49,452 (6.61)	3.31 (2.70, 4.07)	0.000	2.82 (2.29, 3.48)	0.000
Comorbidities						
CVD	2,644 (34.28)	200,198 (26.75)	1.43 (1.36, 1.50)	0.000	1.46 (1.38, 1.54)	0.000
Hypertension	3,207 (41.58)	285,575 (38.15)	1.15 (1.10, 1.21)	0.000	1.18 (1.11, 1.25)	0.000
Diabetes	2,156 (27.96)	140,117 (18.72)	1.68 (1.60, 1.77)	0.000	2.06 (1.96, 2.18)	0.000
CKD	333 (4.32)	35,271 (4.71)	0.91 (0.82, 1.02)	0.104	0.76 (0.68, 0.85)	0.000
Liver disease	111 (1.44)	13,262 (1.77)	0.81 (0.67, 0.98)	0.028	0.81 (0.67, 0.98)	0.028
COPD	617 (8.00)	54,194 (7.24)	1.11 (1.03, 1.21)	0.010	0.98 (0.90, 1.07)	0.674
Rheumatic disease	626 (8.12)	50,033 (6.68)	1.23 (1.14, 1.34)	0.000	1.20 (1.11, 1.31)	0.000
Concomitant Medications						
ACEIs/ARBs	2,254 (29.23)	221,504 (29.59)	0.98 (0.94, 1.03)	0.482	0.81 (0.77, 0.86)	0.000
CCBs	1,209 (15.68)	121,618 (16.25)	0.96 (0.90, 1.02)	0.175	0.83 (0.78, 0.89)	0.000
Diuretics	1,706 (22.12)	131,440 (17.56)	1.33 (1.26, 1.41)	0.000	1.14 (1.08, 1.22)	0.000
Beta-blockers	1,581 (20.50)	144,826 (19.35)	1.07 (1.02, 1.14)	0.011	0.95 (0.89,1.01)	0.108
Antiplatelets/ anticoagulants	2,944 (38.17)	230,959 (30.86)	1.38 (1.32, 1.45)	0.000	1.23 (1.17, 1.30)	0.000
NSAIDs	1,306 (16.93)	119,283 (15.94)	1.08 (1.01, 1.14)	0.017	1.06 (0.99,1.12)	0.076
Polypharmacy (≥ 5 medications)	4,299 (55.74)	378,508 (50.57)	1.23 (1.18, 1.29)	0.000	0.96 (0.91,1.02)	0.163

ADR: Adverse drug reaction. CVD: Cardiovascular Disease. CKD: Chronic kidney disease. COPD: Chronic obstructive pulmonary disease. ACEI: Angiotensin converting enzyme inhibuitor. ARB: Angiotensin receptor blocker. CCB: Calcium channel blocker. NSAID: Non-steroidal anti-inflammatory drug.

**Table 3:** Risk factors of antithrombotic drugs-related ADRs consultations.

Variables	Patient with	Patients without ADR consultation	Crude Odds Ratio (95% CI)	P-value	Adjusted Odds Ratio (95% CI)	P-value
	ADR consultation					
	N = 4,133 (100%)	n = 617,384 (100%)				
Sex (Male)	2,150 (52.02)	334,252 (54.14)	0.92 (0.86, 0.98)	0.006	0.84 (0.79, 0.90)	0.000
Age (Mean ± SD)	68.78 (11.86)	62.54 (15.10)				
< 40 years	51 (1.23)	49,329 (7.99)	Reference			
40 ≤ age ≤ 49	193 (4.67)	62,306 (10.09)	2.30 (2.20, 4.08)	0.000	2.40 (1.76, 3.28)	0.000
50 ≤ age ≤ 59	596 (14.42)	116,850 (18.93)	4.93 (3.71, 6.57)	0.000	3.55 (2.66, 4.74)	0.000
60 ≤ age ≤ 69	1,125 (27.22)	168,984 (27.37)	6.44 (4.86, 8.53)	0.000	4.48 (3.37, 5.94)	0.000
70 ≤ age ≤ 79	1,288 (31.16)	136,262 (22.07)	9.14 (6.91, 12.10)	0.000	5.91 (4.45, 7.85)	0.000
≥ 80 years	880 (21.29)	83,663 (13.55)	10.17 (7.67, 13.50)	0.000	6.15 (4.62, 8.18)	0.000
Comorbidities						
CVD	2,423 (58.63)	192,858 (31.24)	3.12 (2.93, 3.32)	0.000	2.93 (2.74, 3.13)	0.000
Hypertension	1,576 (38.13)	195,491 (31.66)	1.33 (1.25, 1.42)	0.000	1.10 (1.02, 1.18)	0.017
Dyslipidaemia	486 (11.76)	54,140 (8.77)	1.39 (1.26, 1.52)	0.000	1.25 (1.13, 1.38)	0.000
Diabetes	590 (14.28)	80,351 (13.01)	1.11 (1.01, 1.21)	0.016	1.30 (1.19, 1.43)	0.000
CKD	216 (5.23)	27,081 (4.39)	1.20 (1.05, 1.38)	0.009	0.86 (0.75, 1.00)	0.044
Liver disease	56 (1.35)	10,148 (1.64)	0.82 (0.63, 1.07)	0.146	0.84 (0.65, 1.10)	0.209
COPD	504 (12.19)	50,782 (8.23)	1.55 (1.41, 1.70)	0.000	1.23 (1.11, 1.35)	0.000
Rheumatic disease	356 (8.61)	41,664 (6.75)	1.30 (1.17, 1.45)	0.000	1.10 (0.99, 1.23)	0.087
Concomitant Medications						
ACEIs/ARBs	1,220 (29.52)	165,097 (26.74)	1.15 (1.07, 1.23)	0.000	0.75 (0.70, 0.82)	0.000
CCBs	747 (18.07)	86,232 (13.97)	1.36 (1.25, 1.47)	0.000	1.08 (0.99, 1.17)	0.098
Diuretics	1,085 (26.25)	118,951 (19.27)	1.49 (1.39, 1.60)	0.000	1.10 (1.02, 1.19)	0.013
Beta-blockers	1,311 (31.72)	155,582 (25.20)	1.38 (1.29, 1.47)	0.000	1.13 (1.05, 1.22)	0.001
Lipid lowering drugs	486 (11.76)	54,140 (8.77)	1.39 (1.26, 1.52)	0.000	0.88 (0.82, 0.94)	0.000
NSAIDs	680 (16.45)	107,669 (17.44)	0.93 (0.86, 1.01)	0.096	0.95 (0.87, 1.03)	0.224
Polypharmacy (≥ 5 medications)	2,825 (68.35)	364,215 (58.99)	1.50 (1.41, 1.60)	0.000	1.16 (1.08, 1.25)	0.000

ADR: Adverse drug reaction. CVD: Cardiovascular Ddsease. CKD: Chronic kidney disease. COPD: Chronic obstructive pulmonary disease. ACEI: Angiotensin converting enzyme inhibuitor. ARB: Angiotensin receptor blocker. CCB: Calcium channel blocker. NSAID: Non-steroidal anti-inflammatory drug.

## Discussion

This study found that the prevalence of consultations related to cardiovascular drugs ADRs in primary care was 10.60 (95% CI. 10.46, 10.75) per 1000 patients. Statins, beta-blockers, ACEIs/ARBs, and aspirin were the most frequently associated drug classes in ADRs. In addition, this study identified several risk factors for ADRs consultations related to different therapeutic classes.

The findings from this study resonate the results from previous studies using similar method [17, 18, 28–32]. A previous study in Spain primary care showed that the prevalence of consultations related to cardiovascular drugs ADRs was 10.01 per 1000 persons, relatively similar with our finding [30]. Eguale et al found that the cardiovascular drugs-related ADRs rate was 19.1 per 1000 person-years [29]. Focusing on the elderly population, Veehof et al found that the prevalence of antihypertensive-related ADRs in general practice consultations was 11.00 per 1000 persons [31]. Prompt recognition of ADRs in primary care are of importance to facilitate future treatment decisions and reduce the burden of ADRS for patients [17, 33, 34].

In this study, the trend of consultations related to cardiovascular drugs ADRs notably increased in 2004 (0.72%, 95% CI. 0.70, 0.74). Implementation of the Quality and Outcomes Framework (QoF) started this particular year, which partly explained the sharp increase in the ADRs consultations. Under this scheme, general practices in the UK have been incentivised for delivering quality care and monitoring, including for patients with cardiovascular medication use [35, 36]. The increase in the recording of comorbidities and other clinical domains have also been observed in other therapeutic areas such as diabetes and psychiatric disorders [37, 38]. However, the trend decreased in 2010. Previous review on general practitioner’s view on the impact of incentive scheme in improving quality care in the UK showed conflicting results, showing that such scheme may improve patient satisfaction and chronic disease management, but increased pressure for healthcare professionals may hinder its implementation over time [39].

It is not surprising that in this study, the elderly patients (aged 70–79 years) had the highest occurrence of ADRs related to cardiovascular drugs. Altered pharmacokinetics due to physiological changes, comorbidities, and concomitant medication use increase the risk of ADRs among this population [40, 41]. However, this study found that patients aged ≥ 80 years had slightly lower occurrence of ADRs, indicating ‘depletion of susceptible phenomenon’, i.e., the modification ADRs risk in certain population due to several factors, including the past ADRs events and the use of low dose of medication due to age-related conditions [42].

In this study, statins, beta-blockers, ACEIs/ARBs, and aspirin were the most frequently implicated medication classes in the ADRs consultations, comparable with previous reports using UK general practice research databases [18, 32]. Statins and ACEIs are among the most dispensed medications in England, indicating the high importance of monitoring the safety of these medications [5]. Although generally well tolerated, muscle symptoms may occur in a subset of statin users, particularly in those using high intensity regimen [43, 44]. The comparative safety profile between lipophilic and hydrophilic statins has been frequently discussed [45–47]. Muscle symptoms have been reported with all statins, but hydrophilic statins, e.g., rosuvastatin, pravastatin, were considered to have a lower risk of myotoxicity due to reduced non-selective diffusion into extrahepatic tissues, such as muscle cells, while lipophilic statin, e.g., simvastatin, had a higher risk of muscle symptoms [48]. Nevertheless, it is important to consider dose-response relationship and lipid goal attainment when selecting alternate statin for those intolerance to a specific statin [49].

The second most involved drug in the ADRs consultations was beta-blockers. Beta-blockers are a vital component for the treatment of heart failure and myocardial infarction. Beta-blockers have no longer been the first-line choice for antihypertensive treatment due to inferior cardiovascular protection, particularly against stroke compared to RAAS inhibitors and CCBs [50]. Lack of efficacy of beta-blockers in uncomplicated hypertension is attributed to their poor tolerability resulting from various ADRs, such as respiratory symptoms, lethargy, erectile dysfunction, sleep disturbance, blurring of vision, and Raynaud’s phenomenon [51]. A previous trial showed that the number of patients who discontinued beta-blocker due to severe ADRs was double that of diuretic users [52]. Cardio-selective beta-blockers, e.g., bisoprolol and atenolol, are increasingly used due to lower risk of adverse respiratory effect than non-selective beta-blocker [53]. Given its benefit for several conditions, such as heart failure, cardiac arryhtmias; cardio-selective beta-blockers should not be withheld from patients with mild to moderate reactive airway disease [54]. Due to their permeability to cross blood brain barriers, beta-blockers may also causes psychiatric symptoms [55, 56], thus this medication class should be used carefully in patients with neuropsychiatric disorders [57].

This study found that ACEIs and ARBs, first line hypertension treatment, were the third most common implicated drug classes in the ADRs consultations in primary care. Persistent dry cough, gastrointestinal symptoms, hyperkalaemia, hypotension, and dizziness are common ADRs associated with ACEIs/ARBs [58–60]. A previous review showed that ARBs had better tolerability than ACEIs, with 28% lower risk of drug discontinuation due to ADRs [61]. Similar finding was observed in a recent multinational network cohort study which showed significantly lower risk of angioedema, cough, pancreatitis, and gastrointestinal bleeding among ARBs users, with comparable long-term cardiovascular protection with the former [62]. As generic medications for both classes are currently available, the cost may no longer pose a barrier for initial treatment selection [63]. Nevertheless, clinical guideline suggested that ARBs should be reserved for individuals who are intolerant to ACEIs, as the evidence supporting the effectiveness of ACEIs is more robust [22]. Benefit-risk analysis based on individual circumstances should be carefully performed to ensure that hypertension treatment goals can be achieved safely.

The previous history of an established CVD, advanced age, and comorbidities, e.g., diabetes, hyperlipidaemia, consistently appeared as the strongest risk factors for cardiovascular drug-related ADRs consultations. Clinical guidelines generally recommend increased dose/intensity regimen for CVD secondary prevention treatment, which may be associated with more safety events [64, 65]. The presence of comorbidities was also associated with higher number of medications, potentially leading to more ADRs [66]. The use of drug safety alerts to assist healthcare providers during prescribing to prevent ADRs was shown to be effective in reducing ADRs in high-risk population [67]. Nevertheless, a previous review showed that alert fatigue, which refers to clinicians dismissing alerts due to an excessive number of warnings, was prevalent, with 46–96% of drug safety alerts were overridden [68]. Thus, further refinement may be needed, e.g., by re-evaluating clinically relevant alerts to reduce noise; and improving information content to reduce the burden of workflow disruption to effectively reduce the risk of ADRs for patients [68, 69].

### Implications for practice and research

This study found that the burden of ADRs related to cardiovascular drugs in primary care was considerable. As cardiovascular-related conditions, such as hypertension and dyslipidaemia, are largely managed in primary care [20], primary care providers are encouraged to be vigilant in preventing ADRs of these medications to prevent further iatrogenic complications, e.g., by monitoring patients about their concerns and barriers related to medication use, educating patients about early recognition and management of potential ADRs, strengthening medication review system particularly for high-risk patients, and performing adequate ADRs documentation/recording to facilitate future treatment decisions [17, 70–73]. A previous study showed that patients who received prior information on potential ADRs from their health care professionals were less likely to report drug complications and were more satisfied with their treatment [70]. In addition, it is recommended to implement more rigorous clinical and medication adherence monitoring for patients affected by ADRs as our previous studies showed that patients who experienced ADRs related to statins and ACEI/ARB had an increased risk of subsequent cardiovascular events compared to those who did not experience ADRs [7, 8]. These previous studies are different with current study, as these studies are cohort studies investigating the subsequent treatment outcomes following the ADRs, while current study focused on the prevalence and risk factors of ADRs.

Further research investigating the effectiveness of intervention to improve early detection and management of ADRs in less-controlled healthcare environment such as primary care setting, may be needed. An electronic patient-centred platform where patients could discuss and report the problem of their medication to health care professionals has the potential to improve medication safety and treatment outcomes [74].

### Strengths and limitations

This study provides information on the burden and temporal trend of consultations related to cardiovascular drugs ADRs in UK primary care. Validated designated codes were used to identify ADR consultations in primary care database, which represent 6% of the UK population [18, 23, 32]. However, this study also has several limitations. Database screening method may be prone to under-reporting due to variability of ADRs assessment and/or reporting by primary care providers. Despite the difference in the ADRs prevalence observed (2.4% versus 9.0%), a previous comparative study showed that administrative database screening has advantages compared to manual chart review, considering high positive predictive value obtained (87.6%) and reduced resource utilised (two-person hours versus 35 person-hours) for ADRs detection, thus a larger population can be included [75]. This study may serve as a basis for further investigation on the burden of ADRs in the population.

## Conclusions

This study showed that the burden of cardiovascular drug-related ADR consultations in primary care was considerable. Statins, beta-blockers, ACEIs/ARBs, and aspirin were the most frequently implicated cardiovascular drug classes in the ADRs consultations. More rigorous clinical monitoring should be performed for patients affected by ADRs to mitigate the risk of suboptimal treatment outcomes.
